# Lower Limb Biomechanics during the Topspin Forehand in Table Tennis: A Systemic Review

**DOI:** 10.3390/bioengineering9080336

**Published:** 2022-07-25

**Authors:** Yuqi He, Gusztáv Fekete, Dong Sun, Julien S. Baker, Shirui Shao, Yaodong Gu

**Affiliations:** 1 Faculty of Sports Science, Ningbo University, Ningbo 315211, China; heyuqi0809@outlook.com (Y.H.); sundong@nbu.edu.cn (D.S.); 2 Faculty of Engineering, University of Pannonia Veszeprem, 8200 Veszprém, Hungary; 3 Savaria Institute of Technology, Faculty of Informatics, Eötvös Loránd University, H9700 Szombathely, Hungary; fg@inf.elte.hu; 4 Department of Sport and Physical Education, Hong Kong Baptist University, Hong Kong, China; jsbaker@hkbu.edu.hk

**Keywords:** kinematics, kinetics, table tennis, topspin forehand, muscle activity

## Abstract

The aim of this study is to review the valuable lower limb biomechanical contribution to table tennis topspin forehand. Databases included Scopus, PubMed, and Web of science. In this case, 19 articles were selected for the systematic review. The mechanics of the plantar, lower limb joints kinematics and kinetics, muscle activity, and racket-joint relationship are described through gender, performance level, and footwork. The study found that the hip movement characteristics and the hip muscle group activity following a proximal-to-distal sequence strategy significantly contributed to the maximum acceleration of the racket. Optimizing the motion strategy of the ankle and plantar as well as the ankle muscle group activity is beneficial for the transmission of energy in the kinetic chain. Muscle groups around the ankle and subtalar joints are heavily activated during landing to maintain foot stability during the landing phase. Lower limb muscle development plays an important role in movement control and stability as well as sports injury prevention in table tennis footwork during the performance of the topspin forehand. Furthermore, physical development levels and anatomical differences (such as hip and lower trunk muscle strength differences), maybe the main reasons for gender differences observed during the topspin forehand. Systematically summarizing this valuable information can contribute to athletes’ and coaches’ knowledge to enhance topspin forehand performance and training regimes. We suggest that future research could consider the joint contact forces, ball movement, and ball-racket impact during a performance of topspin forehand.

## 1. Introduction

Table tennis is a spectator and highly competitive sport which have the characteristics of high speed and fast rotation. Athletes perform perfect strokes in combination with upper and lower limb movement, handwork, footwork, and trunk rotation. This is in combination with racket movement and positioning in a limited period to pose an attacking threat to the opponent. As one of the most popular racket sports, table tennis attracts over 40 million participants around the world [1,2]. These include athletes, researchers, and coaches. The massive growth in participants has also contributed to the sport’s technological iteration. Meanwhile, with the development and support of modern science and technology, table tennis playing skill has been optimized and the intensity of competition has also been further enhanced. To maintain excellent sports performance, athletes and coaches need to fully grasp and understand the internal mechanisms of table tennis technology.

Topspin forehand is known as one of the most basic and aggressive strokes in table tennis. Especially for an elite offensive player, excellent forehand topspin skill is necessary to maintain a strong attacking posture [3]. Some clinical experimental studies have collected kinematic and dynamic information about players hitting forehand topspin through 3d motion capture systems, such as infrared cameras and high-speed cameras. The whole-body coordination mechanism is very important in table tennis, and the performance level of the upper limbs is largely determined by the lower limbs [4,5]. In recent years, the important role of lower limb function in table tennis has been widely studied and reported [2,4,6,7,8,9,10,11]. As the origin of the kinetics chain, perfect lower limb movement performance will benefit the velocity of the racket and ball [4,12,13]. Although several studies investigated biomechanical information or highlighted the lower limb during topspin forehand, their experimental design, protocol process, and methods were generally inconsistent. Meanwhile, the common characteristics of elite athletes with the same skill and playing mode can reflect the internal mechanisms of sports at different levels and the technical characteristics. Therefore, to optimize topspin training items and provide guidance information, it is necessary to explore the common lower limb biomechanical characteristics of high-level athletes during topspin forehand strokes.

Biomechanics reviews have provided strong support for improving performance and preventing injury in various sports [12], such as tennis [14,15] and football [16,17]. However, as we know, there are few table tennis biomechanics reviews. Recently, there was a table tennis biomechanics review that investigated the movement maneuvers and playing levels [12]. The significance and originality of this study were that it explores the valuable biomechanics evidence of the lower limb during topspin forehand skill in table tennis. In addition, the application of this study was to provide specific biomechanical information to the researchers who focus on the field of the lower limb or table tennis topspin forehand. Hence, this study provides a systemic review of the evidence of lower limb kinematics, kinetics, muscle activity, and plantar pressure character during the topspin forehand.

## 2. Materials and Methods

### 2.1. Protocol Registration

This study was conducted according to preferred reporting items for PRISMA (systematic reviews and meta-analysis) statements [18]. The protocol of this systematic review was registered on INPLASY. (Registration number: INPLASY202260096).

### 2.2. Search Strategy

To ensure the accuracy of the study results, the design of the search was checked and approved by all the authors of the study. The electronic databases of ISI Web of Science, Scopus, and PubMed were used for searching electronic literature from the earliest available date to 7 July 2022. The search strategies were shown in Table 1.

### 2.3. Eligibility Criteria

The following inclusion criteria were used to screen the studies (1) the article should be published in English; (2) the article should be published in a peer-review journal; (3) biomechanics research with a table tennis experiment process; (4) the article investigated the lower limb biomechanics information of topspin forehand of table tennis players.

Articles were excluded if (1) the participant was under 18 years old; (2) had a musculoskeletal problem, injury, or rehabilitation; (3) the article focused on table tennis robots or machine learning; (4) the article only used theoretical model or simulations; (5) without specified stroke movement information.

### 2.4. Study Risk of Bias Assessment

Two reviewers (Y.H. and Y.G.) independently screen the methodological quality by including articles based on the Combie criteria which included seven domains [19]. According to the Combie evaluation tool, the total score of each article was 7.0 points, and the quality degree of articles was defined as A, B, and C which indicated 6.0~7.0 points, 4.0~5.5 points, and 0~4.0 points, respectively. Any disagreements arising in the quality assessment would be resolved by an independent arbitrator (G.F.).

### 2.5. Data Extraction and Management

As shown in Table 1, two reviewers (Y.H. and Y.G.) independently collected the data from all selected studies based on the participant, intervention, comparisons, and outcomes principle (PICOS) with a standard form.

## 3. Results

### 3.1. Literature Selection

**Figure 1:**
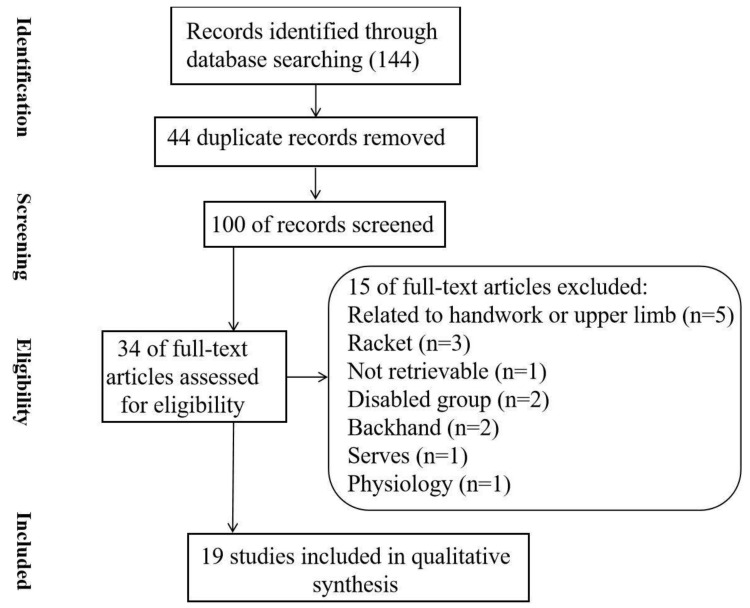
Flowchart of the systematic search and selection process.

There were 144 articles included in the initial search. After the process of removing duplicates and pooling, there were 100 articles selected for the screening section. Totally 34 studies were finally selected in the process of eligibility (Figure 1). In the eligibility process, articles were excluded if they were focused on the handwork (*n* = 5), racket (*n* = 3), backhand (*n* = 2), serves (*n* = 1), and the physiology issues (*n* = 1), and the participants with disabilities (*n* = 2). Furthermore, one article could not be retrieved. Finally, 19 articles were selected for the systematic review.

### 3.2. Original Characteristics

**Figure 2:**
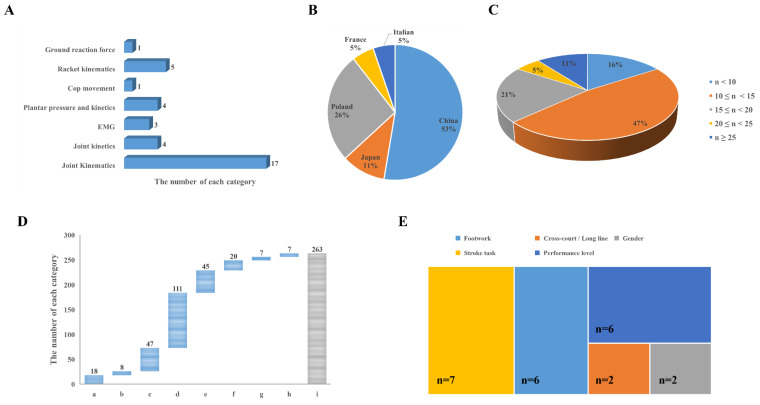
Characteristic information: (**A**) parameters; (**B**) country of included studies; (**C**) sample size; (**D**) performance level of player, “a” refers to university level, “b” refers to national Ⅲ, “c” refers to national II, “d” refers to national I, “e” refers to the national team level, “f” refers to national top 16, “g” refers to national top 10, “h” refers to national top 200, “I” refers to total; (**E**) maneuvers type.

The original characteristics of each included study could be seen in Figure 2 and Table 2. The parameters of joint kinematics were most focused which included 17 studies. The racket and plantar information were also focused which included 5 and 4 studies, respectively. The percentage of included studies from China and Poland was 53% and 26%, as well as Japan, Italian, and France were 11%, 5%, and 5%, respectively. There are 47% of the studies’ sample size was in the 10 to 15, and the total sample size of included studies was 263, a total of 111 players’ performance levels was belonging to the national I. The stroke task, footwork, and performance level were the most concerned maneuvers setting which included a total of 7, 6, and 5 studies, respectively. 

### 3.3. Risk of Bias

The quality of all selected studies was assessed in terms of risk of bias, and the results were presented in Table 3. There are 79% of the included studies scored better than 5.5 points in which defined quality A, and there are 4 articles scored less than 6.0 points in which defined quality B. The quality of the selected literatures in this study was indicated high and moderate.

### 3.4. Gender in the Topspin Forehand

Two articles reported on the gender differences in forehand topspin, one of which explored the effect of gender on lower limb joints biomechanical characteristics during the forehand topspin stroke with chasse step footwork [7]. Compared with female athletes, male athletes performed significantly greater movements in the lower limb joints, such as the extension and flexion of the hip, trunk, and knee joints [7,22]. However, In the backward phase, female athletes showed a significantly great hip abduction than male athletes. The maximal acceleration of the playing hand of the male athletes was significantly greater than the female athletes during the topspin forehand [22].

### 3.5. Performance Level in the Topspin Forehand

A total of 6 articles investigated the biomechanical characteristics of the lower limbs of different performance levels athletes during the topspin forehand. Five reported the kinematic information of the lower limb joint, two were related to footwork, one was related to electromyogram (EMG), and one was related to the movement of the center of pressure (COP). In this study, the advance player (AP) and intermediate player (IP) were defined according to the selected articles in which the advance player refers to the national I level and the intermediate player refers to the national II level. Lower trunk axial rotation of AP contributed more to racket speed during the topspin forehand [3]. Lower limb joint angles, joint velocity, and range of motion were greater in the sagittal and horizontal planes in AP, such as the hip [6,23], knee [23], and ankle [2] joints. In addition, the movement characteristics of COP were also significantly different among athletes of different levels. Compared with IP, the AP showed greater medial-lateral COP displacement during the backward phase, but less anterior-posterior displacement throughout the stroke process [24]. In addition, the AP have a larger plantar contact area than IP [6]. Results indicated that AP possessed better foot drive skills and ability of foot movement control during the topspin forehand [6,20,24].

### 3.6. EMG in the Topspin Forehand

Three articles addressed EMG information during the topspin forehand, two were based on table tennis footwork, and two were based on performance level differences. Lower limb muscle activity levels were significantly higher during forehand topspin compared with other types of strokes [10]. Hip, knee, and ankle flexion muscle groups such as the biceps femoris, gluteus maximus, rectus femoris, gastrocnemius, and soleus were thoroughly activated during high-intensity topspin forehand strokes [10,23].

### 3.7. Footwork in the Topspin Forehand

A total of 6 articles explored the biomechanical characteristics of footwork during the topspin forehand. The lower limb biomechanics of cross-step, chasse step, and one-step footwork seem to have received more attention and research. The chasse step footwork is a side movement that could combine with racket movement to perform offensive and defensive strokes in table tennis [11,28]. Comparing the long-distance chasse step footwork with the short-distance chasse step footwork, the ankle joint ROM and angular velocity in the coronal and transverse planes of the long-distance chasse step footwork were significantly faster than the short-distance chasse step footwork during the topspin forehand [28]. The maximal knee flexion and ankle inversion angular velocity of the cross-step footwork were significantly greater than the chasse step footwork during the topspin forehand [5]. The joint angles and ROM of the hip, knee, and ankle joints of the one-step footwork were significantly smaller than those of the cross-step and chasse step footwork [5]. Gender and level factors were also important in relation to research content in footwork biomechanics during the topspin forehand. In the foreword phase, hip angular velocity and ROM in the male athlete were significantly greater than the female athletes [7]. Compared with IP, the AP showed significantly greater flexion velocity in the hip and knee during cross-step footwork, as well as significant hip and knee moment during a fast topspin forehand using the cross-step footwork [23].

### 3.8. Plantar Biomechanics in the Topspin Forehand

A total of 4 articles explored the mechanical characteristics of the plantar during the topspin forehand. Plantar mechanics are related to lower limb drivability. In addition to the movement of COP [24], indexes such as pressure in various plantar regions [5,11,20], plantar force [11], contact area [6], force-time integral [11], and pressure-time integral [5,11] have been successively studied and reported. Overall, the differences in plantar mechanical characteristics were concentrated in the first metatarsal, the medial-lateral of the forefoot, and the medial-lateral of the rearfoot [5,6,11,20]. The peak pressure in the total foot and toe regions of the cross-step and chasse step footwork were significantly greater than that in the one-step footwork [5]. The peak pressure in the total foot and first metatarsal regions was significantly greater in the cross-step than in chasse step footwork [5,11]. Chasse step footwork showed significantly greater plantar force, force-time integral, and pressure-time integral than one-step footwork in both backward and forward phases. In the foreword phase, the peak pressure in the toe region of chasse step footwork was significantly greater than that of one-step footwork [5,11]. Differences in performance levels also led to differences in plantar mechanics, with AP exhibiting higher peak pressures in the medial-lateral forefoot region and the medial-lateral rearfoot region when performing chasse step footwork [20]. In addition, AP have significantly larger plantar contact areas during topspin forehand [6].

### 3.9. Relationship between Lower Limb Joints and Racket in Topspin Forehand

Five articles reported the relationship between lower limb joints biomechanical characteristics and racket movement during the topspin forehand. The influence of the human joint movement on racket movement has always been the main content of biomechanical research on the topspin forehand. The maximum speed of the racket is increased through the human kinetic chain effect, which brings benefits to enhancing the rotation and aggression of the ball [5,6,9,23]. In general, racket velocity is related to the angular velocity of axial motion of the hip, pelvis, and ankle joints. Specifically, the flexion angular velocity of the hip joint on the playing side and the extension angular velocity of the other side [25,26], and the plantar flexion angular velocity of the ankle joint during topspin forehand [2]. The peak velocity of pelvic axial rotation and the work carried out by the pelvic axial rotation torque on the playing side has a positive correlation with the horizontal velocity of the racket at impact during the topspin forehand [26].

## 4. Discussion

Research on the biomechanics of the lower limbs in table tennis has received extensive attention and reports. The key information and findings of the research have been extracted, categorized, combined with practice, and applied for training and participation in table tennis competition. This study provides a systematic review of lower limb biomechanical studies on forehand topspin skills. Information on joint kinematics, muscle activity, joint kinetics, plantar dynamics, and synergistic relationships between racket and joints during topspin forehand. The review summarizes and considers footwork, gender, and performance level. The purpose was to reveal the inner mechanisms of the lower limb biomechanics during topspin forehand skill, and to provide a theoretical basis and reference for scholars and related practitioners. In addition, the paper also outlines unsolved problems and provides possible solutions.

Personalized training is one of the principles of sports training, which aims to adapt training programs, training methods, and training loads to the individual needs of the athlete [6]. Athletic diversity is often attributed to factors such as body anatomy, level of motor skill development, gender, level of technical performance, age, and psychological quality [22]. The consequences of these factors are reflected in athletic performance, forming the movement characteristics of joints and muscles. Gender research is limited around lower limb biomechanics in table tennis. Existing studies have shown that male athletes’ hip and knee joints have more sports participation and contribution in the stroke phase of the topspin forehand, and female athletes have more sports participation and contribution of the upper limb joints. A possible reason for this finding is that male athletes have stronger hip and knee flexion muscle groups (such as gluteus, anterior thigh muscles, and back thigh muscles) than female athletes and can produce stronger contractions. Axial rotation of the trunk is primarily produced by the muscle groups around the hip joint [31], movements of the hip joint and pelvis have a positive relationship with racket acceleration [26], which also explains that the maximum swing acceleration of male athletes is significantly greater than that of female athletes. Therefore, in the forehand topspin skill, the underlying reasons for gender differences may be mainly differences in the level of physical function and anatomy. Compared with male athletes, the topspin forehand skill of female athletes relies more on the rotation of the elbow and shoulder joints [22], which may lead to the more agile and flexible technical style of female athletes. When exploring the factors of performance levels during the topspin forehand, in addition to differences in racket acceleration caused by differences in physical development levels, the mastery of motor skills, and the coordination of body joints (such as power generation and energy transfer in the kinetic chain) are potential influences. The AP have a shorter stroke time [1,2,6,9], and they provide rapid hip flexion/extension angular velocity through the rapid work of the muscles around the hip joint [10,23,31], which enhances the axial rotation of the lower trunk and increases the acceleration of the racket [26,32]. The shorter stroke time is beneficial to the athlete with sufficient time to prepare for the next stroke and execute the strategy [3,9]. This may also explain why AP had significantly greater lower limb joint movement in both the sagittal and transverse planes. Previous studies have shown that when performing high-intensity forehand topspin, the lower body muscles of athletes are fully activated, and the muscle activity is significantly higher than that of other forms of hitting [10]. This demonstrates the involvement and contribution of lower body muscles in the forehand topspin stroke. Therefore, we strongly recommend building strength and explosiveness of the lower limb muscles, as excellent proficiency optimizes the transmission efficiency of the kinetic chain. In addition, long hours of practice in forehand topspin skill are also necessary. Based on the stretching-shortening cycle theory (SSC), the elastic energy stored in the muscle-tendon stretching phase can enhance the concentric movement of the muscle, and the training of SSC and strength should be combined to performed which could ensuring that athletes are technically competent at each phase prior to progress in strength and complexity [33]. Further to this, strong lower body strength can bring gains to the stability and motor control of footwork and provide support for the stability of the backward phase. He et al. [9] reported the important role of the ankle joint during the forehand topspin, strengthening the muscles around the ankle joint and the subtalar joint can help athletes maximize the important role of the foot as the origin of the kinetic chain.

The foot and plantar biomechanical characteristics of forehand topspin have been extensively studied and reported in recent years, and this information is generally considered to be related to lower limb drive ability [6,20,24] and the origin of the kinetic chain [9,29]. Peak pressure, plantar force, COP displacement, COP velocity, contact area, are the basic parameters of plantar biomechanical research. During the backward phase, the medial-lateral displacement of the COP was significantly greater in the AP, which may imply more aggressive and active lower limb movement in AP. The anterior-posterior displacement of the COP was significantly smaller in both the backward phase and forward phases, which may imply a more stable center of gravity. Compared with the forward phase, one of the main functions of the foot in the backward phase is to maintain stability and provide stable preparation conditions for the hitting action. He et al. [11] investigated the peak pressure at the LR and MR of the one-step footwork during the backward phase were significantly great than the chasse step footwork. However, there was no significant difference in the study of Lam et al. [5]. A possible explanation is that the flight velocity and angles of the table tennis balls in these two studies were not unified, nor were the initial positions and initial movements of the players unified, thus resulting in different plantar pressure characters during foot landing of the backward phase. The study of plantar pressure characteristics of footwork during the topspin forehand not only helps us to understand the important role of the foot in the forehand topspin movement but also helps to further understand the internal mechanism of the kinetic chain generation and transmission [6,11,20,24] and can also prevent possible stress injuries [5,11], as well as provide theoretical support for the research and development of table tennis shoes [5,11]. The combination of chasse step footwork and cross-step footwork with forehand topspin skills is very common in table tennis. The angular velocities of knee flexion and ankle inversion were significantly higher in the cross-step footwork than in the chasse step footwork, and the peak pressure in the first metatarsal region was significantly greater than that in the chasse step footwork. A possible explanation is that the athlete follows a proximal-to-distal sequential strategy that utilizes whole-body weight transfer to accomplish better energy transfer in the kinetic chain, bringing a gain to the racket’s maximum velocity [5,25,29,34]. However, this also means an increased risk of injury. 

There are several table tennis review studies that have to be mention, and it is valuable to compare was this study. Wong et al. [12] summarized the biomechanics evidence of table tennis strokes and reported the full body information during different strokes type based on performance level and maneuvers in table tennis. Ferrandez et al. [35] summarized the studies of biomechanics, physiology, and injuries in table tennis. They investigated the understanding of energy generated by footwork which contributes to racket velocity was necessary and interesting. This point was further supported and discussed by this study. There are a few other types of racquet sports review studies of lower limbs biomechanics that need to be mention also. Lam et al. [36] summarized the biomechanics studies of lower limbs in badminton luges, and they report the motor control strategies of knee and ankle joint, the plantar loading as well as the possible design information of badminton shoes. Genevois et al. [37] review the mechanics description studies of backhand performance in tennis, they reported the difference between the onehanded and twohanded backhand in motor coordination as well as they pointed the revelation biomechanics information of gender and age was necessary to focus on in the further research. 

In this field of research, some unanswered questions need to be raised. Firstly, in the existing biomechanical studies on topspin forehand skills in table tennis, there is little research and motion capture of the ball movement. In fact, combining human motion with ball motion to explore the inner connection between human movement and ball movement can further elaborate and develop the depth and breadth of research in this field. Secondly, the current research also lacks reports on the collision effect between the ball and the racket, which is actually an important section of research. Thirdly, from the perspective of sports injuries, it is also interesting and valuable to use musculoskeletal models to further explore the biomechanical characteristics of joints, such as calculating joint contact and shear forces through Opensim software.

The present study could probably provide some support for clinical application. The table tennis coach and the professorial athlete could acquire valuable information to optimize training strategy and enhance the lower limbs’ motor control ability during topspin forehand. Relevant researchers could quickly establish a basic understanding and knowledge base on the lower limb biomechanics of table tennis topspin forehand through this study.

## 5. Limitations

There are some limitations in this study. (1) The included articles of this study hardly report effect size and statistical power in detail, and there are certain statistical limitations, and follow-up research can be strengthened. (2) The existing research results are all derived from the experimental environment rather than the real game environment, which is the main limitation. (3) The existing researchers rarely observe the motion of the ball and the collision effects between the ball and the racket. These are research directions worthy of further attention. (4) Existing studies have not studied the contact force of the lower limb joints in forehand topspin and understanding the joint contact force can help predict possible joint damage. Therefore, we suggest that future research could consider the joint contact forces, ball movement, and ball-racquet collision effects during the performance of topspin forehand.

## 6. Conclusions

In general, the lower limb biomechanics for forehand topspin skills are widely studied. The mechanics of plantar pressure, lower limb joints kinematics and kinetics, muscle activity, and racket-joints relationship are described through gender, performance level and footwork. The hip muscle group are activated first, following the proximal-to-distal sequence to drive the hip, knee, and ankle joints to perform correct footwork to reach the perfect position to hit the ball. The medial and lateral rearfoot, 5th metatarsal, and lateral forefoot regions absorb energy from impact during landing, and muscle groups around the ankle and subtalar joints are heavily activated during landing to maintain foot stability during the landing phase. Based on the stretching-shortening cycle theory, the elastic energy stored in the muscle-tendon stretching phase can enhance the concentric movement of the muscle, and the concentric contraction of the muscle group around the subtalar joint drives the foot eversion and plantar flexion, and then, the energy following the kinetic chain transmission effect, the contraction of muscle groups around the hip and knee joints, drives the generation of axial rotation and moment. Axial rotational angular velocities and moments of the hip joint provide gains for the maximum acceleration of the racket, while whole body weight transfer and energy transfer also result in larger loads and peak pressures in the medial region of the first metatarsal, forefoot. Systematically summarizing this valuable information can make a contribution to athletes and coaches to enhance topspin forehand performance and training regimes level. We suggest that future research could consider joint contact forces, ball movement, and ball-racket impact during topspin forehand performance.

## Figures and Tables

**Table 1:** Search strategies in each electronic database.

Database	Search Strategies	Result
PubMed	(“racket sports” [Mesh] AND “table tennis” [All Fields] AND “biomechanics” [All Fields] OR “kinematics” [All Fields] OR “kinetics” [All Fields] AND “topspin” [All Fields] NOT “backhand” [All Fields] AND “lower limb” [All Fields])	7
Web of Science	(“table tennis” AND (“lower limb” AND (“biomechanics” OR “kinematics” OR “plantar pressure” OR “topspin”)))	25
Scopus	(((((“kinematics” OR “biomechanics” OR “kinetics” OR “topspin” OR “gender” OR “plantar pressure”) AND “table tennis”) AND “lower limbs”) AND NOT “backhand”) AND NOT “review”)	112

**Table 2:** The characteristic information of included studies.

Author	Sample Size (Total)	Gender (Male/Female)	Country	Mean Age (Year)	Experience (Year)	Variable	Performance Level	Biomechanical Parameters	Key Findings
Yu et al. (2019) [20]	18	18/0	China	AP (23.5); IP, (22.7)	AP (14.8); IP (0.45)	Performance level/Footwork	University team	Joint kinematics and kinetics; Planar pressure	↑ AP’s forefoot and rear-foot dorsiflexion, hallux plantarflexion; ↑AP’s peak pressure under the lateral forefoot, and angular velocity in the medial and lateral rear-foot during the chasse step
Iino et al. (2009) [3]	17	/	Japan	AP (20.6); IP (20.6)	AP (11.2); IP (7.4)	Performance level	Division I and national level (*n* = 9); Division III (*n* = 8)	Joint and racket kinematics	↑ The AP’ lower trunk axial rotation contributes to the racket speed at impact and the time required for racket acceleration
Malagoli Lanzoni et al. (2018) [21]	7	7/0	Italian	22.2	10.2	CC/LL	Top 200 in Italian	Racket kinematics; Feet-table angle; Angle and moment of the MMV of racket	At the MMV of the racket in LL: ↑ Right knee angles flexion; ↑ Angles between the feet and the table;
Bańkosz et al. (2020a) [22]	12	6/6	Poland	Male (22.9); Female (21.1)	/	Gender/stroke task	National team level	Maximal acceleration of the playing hand; Upper and lower limb kinematic	Male: Use large muscle groups and large joints (hip joints, trunk joints in extension and flexion); The difference in the values of maximal acceleration reached almost 50 m/s2 in topspin forehand (*p* < 0.01) and 20 m/s2 in backhand (*p* < 0.01)
Chen et al. (2022) [23]	20	20/0	China	AP (20.6); IP (20.6)	/	Performance level/Footwork	Division I (*n* = 10); Division II (*n* = 10)	Racket speed; Lower limb kinematics; EMG	AP: ↑ MMV of the racket during FS phase; ↑ Joint angular velocities at the topspin instant; ↑ RMS and EMG integrals of the abdominal external oblique as well as biceps brachii muscles; ↑ Hip flexion/extension and knee flexion torques at fast speed (240° /s);
He et al. (2020) [2]	12	12/0	China	22.5	10.4	CC/LL	National Division I	Lower limb kinematics	DS: ↓ Time during the BS and the FS phases; ↑ Ankle internal rotation and inversion during the BS; ↓ Knee abduction and external rotation during the BS; ↑ Knee extension during the FS; ↓ Hip adduction and knee internal rotation during the FS. SS: ↑ ROM of ankle plantar flexion external rotation; ↑ ROM of knee extension; ↑ knee internal rotation.
Fu et al. (2016) [24]	26	26/0	China	AP (20.1); IP (21.2)	AP (13.4); IP (10.2)	Performance Level	AP: National division I (13); IP: National division II (13)	COP; Lower limb kinematics	AP: ↑ Medial-lateral COP displacement at backward-end; ↓Anterior-posterior displacement at both backward and forward ends; ↑ Ratio of COP velocity Between the forward swing and backswing; Better foot drive technique and ability of foot motion control during forehand
Bańkoszet al. (2018a) [25]	10	0/10	Poland	16.0	/	Stroke task	Top 16 junior players in Poland	Joints angular and racket velocity; Lower limb kinematics	Racket velocity was correlated with angular velocities (hip extension on the playing side; Hip flexion on the opposite side; Ankle flexion) in the case of a topspin forehand performed with maximal force - “heavy” topspin;
Qian et al. (2016) [6]	26	26/0	China	AP (20.1); IP (21.2)	AP (13.4); IP (10.2)	Performance Level	AP: National division I (*n* = 13); BP: National division II (*n* = 13)	Lower limb kinematics; Plantar contact area	AP: ↑ Hip flexion and knee external rotation at BS; ↑ Hip internal rotation and extension at FS; ↑ contact areas at both events; ↑ Joints angular changing rate during FS at the ankle and hip; Better ability of using lower limb drive in forehand.
Yang et al. (2021) [7]	10	5/5	China	Male (21.0); Female (21.0)	Male (14.0); Female (12.0)	Gender/Footwork	National division I	Lower limb kinematic	Male: ↓ Time in the BS and longer in the FS; ↑ Knee external rotation during the BS; ↓ Hip flexion, greater hip adduction and abduction during the entire motion cycle; ↑ Knee external rotation during the BS; ↓ Knee flexion ROM in the BS; ↑ Knee extension ROM in the FS; ↑ Hip flexion and adduction; ↑ Internal rotational velocity of the hip joint in the FS; ↑ Hip internal rotation ROM in the FS; ↓ Hip external rotation ROM in the BS.
Iino (2018) [26]	18	18/0	Japan	20.7	12.2	Stroke task	Division I (*n* = 12); Division II (*n* = 6)	Kinematic and kinetic of racket; Pelvis kinetics	The peak pelvis axial rotation velocity and playing side hip pelvis axial rotation torque were positively related to the racket horizontal velocity;
Lam et al. (2018) [5]	15	15/0	China	23.6	/	Footwork	Division I	Lower-limb kinetics and kinematics; GRF; Plantar pressure	One step: ↑ GRF loading, knee flexion angle, knee moment, ankle inversion and moment; Side-step and cross-step: ↑ Peak pressure was observed in the total foot, toe, 1st, 2nd and 5th metatarsal regions; Cross-step: ↑ Peak pressure in medial midfoot and heel regions than one-step; ↑ Peak pressure in total and 1st metatarsal regions than side-step.
Bańkosz et al. (2020b) [27]	7	7/0	Poland	23.0	/	Stoke task	Top 10 Polish senior athletes	Kinematics	↓ The variability of the acceleration values; ↑ Variability in the angular parameters; ↓ The variability of the acceleration values.
Yu et al. (2019) [28]	12	12/0	China	20.64	12.7	Footwork	National level	Lower limb Kinematics and EMG	In the long chasse step: ↑ The angle change rate of the ankle; ↑ROM in the coronal and transverse planes; ↑ Hip in the sagittal and transverse planes; ↓ hip in the coronal plane; The vastus medialis was the first activated muscle in the chasse step.
Mansec et al. (2017) [10]	14	14/0	France	27.1	/	Stroke task	National level	EMG	↑ EMG amplitude of forehand top and the forehand smash compared with other strokes; Both biceps femoris and gluteus maximus were strongly activated during the smash, forehand spin and forehand top; ↑ activation of vaste and rectus femoris during the forehand spin; ↑activation of gastrocnemii and soleus during the smash
He et al. (2021a) [11]	12	12/0	China	22.0	11.0	Footwork	National level 1	Lower limb kinetic (plantar pressure)	One step: ↑ Plantar force than the chasse step during 6.92%–11.22% BS; ↑ Maximum plantar force in the BS; ↓ Maximum plantar force in the FS; ↑ Peak pressure in the medial rearfoot, lateral rearfoot and lateral forefoot in BS; ↓Force time integral and pressure time integral in BS; Chasse step: ↑ Plantar force during 53.47%–99.01% BS; ↑ Plantar force in 21.06%–84.06% during FS; ↑ Peak pressure in the Toe in FS; ↑ Force time integral and pressure time integral in FS.
Bánkosz et al. (2018b) [29]	10	0/10	Poland	Four juniors (18.0); Six Seniors (24.8)	/	Stroke task	Top 16 in Poland in their age Categories	Upper and lower limb kinematic	Attempt to achieve maximal racket velocity based on the principles of proximal-to-distal sequences and summation of speed with a stretch-shortening character of cycle performing topspin forehand; The essential differences between type of topspin forehand occurred in the ROM; Increased power of topspin shot was accompanied by a significant increase pelvis rotation, and knee flexion
He et al. (2021b) [9]	10	10/0	China	/	AP(10.0); IP (9.0)	Performance Level	AP: National Level I (*n* = 5); IP: National Level II (*n* = 5)	Lower limb kinematic	AP: ↓ Knee and hip flexion in the BS; ↑ Ankle varus and eversion in the BS and FS; ↑ Angular changing rate of ankle dorsiflexion and varus in the BS with ankle plantar flexion and eversion during the FS; ↑ Ankle internal rotation and external rotation in the BS and FS phase; ↑ Ankle dorsiflexion and plantarflexion ROM in the BS and FS phase.
Bańkosz et al. (2020c) [30]	7	7/0	Poland	/	/	Stroke task	Poland’s national team	Lower limb Kinematic; Time Duration; Acceleration of “playing hand”	↓Variability in stroke time duration; ↑ Intra individual variability of angles; ↓Inter individual and intra individual variability of knee and elbow angles; ↓Variability in hand acceleration; Individual players achieved relatively constant hand acceleration at the contact moment

Note: The “AP” and “IP” refers to advance player and intermediate player. The “CC” and “LL” refers to cross-court and long line. The “FS” and “BS” refers to foreward swing and backward swing. The “DS” and “SS” refers to diagonal shot and straight shot. The “MMV”, “EMG”, “ROM”, “COP”, “RMS”, and “GRF” refers to maximum velocity, electromyogram, range of motion, center of pressure, root mean squares, ground reaction force, respectively.

**Table 3:** Study risk of bias assessment.

Studies	➀	➁	➂	➃	➄	➅	➆	Grade	Quality
Fu et al. (2016) [24]	Yes	Yes	Yes	Yes	Yes	No	Yes	6	A
Malagoli Lanzoni et al. (2018) [21]	Yes	Yes	Yes	Yes	Yes	Unclear	Yes	6.5	A
He et al. (2020) [2]	Yes	Yes	Yes	Yes	Yes	No	Yes	6	A
Chen et al. (2022) [23]	Yes	Yes	Yes	Yes	Yes	No	Yes	6	A
Le Mansec et al. (2017) [10]	Yes	Yes	Yes	Yes	Yes	Unclear	Yes	6.5	A
Yu et al. (2019a) [28]	Yes	Yes	Yes	No	Yes	Unclear	Yes	5.5	B
Lam et al. (2019) [5]	Yes	Yes	Yes	Yes	Yes	Yes	Yes	7	A
He et al. (2021a) [11]	Yes	Yes	Yes	Yes	Yes	No	Yes	6	A
Yang et al. (2021) [7]	Yes	Yes	Yes	Yes	Yes	No	Yes	6	A
Bankosz et al. (2020a) [22]	Yes	Yes	Yes	Unclear	Yes	No	Yes	5.5	B
Yu et al. (2019b) [20]	Yes	Yes	Yes	No	Yes	Unclear	Yes	5.5	B
Qian et al. (2016) [6]	Yes	Yes	Yes	Yes	Yes	No	Yes	6	A
He et al. (2021b) [9]	Yes	Yes	Yes	Yes	Yes	No	Yes	6	A
Bankosz et al. (2020b) [27]	Yes	Yes	Yes	No	Yes	No	Yes	5	B
Bankosz et al. (2018a) [29]	Yes	Yes	Yes	Yes	Yes	No	Yes	6	A
Bankose et al. (2020c) [30]	Yes	Yes	Yes	Yes	Yes	No	Yes	6	A
Iino et al. (2009) [3]	Yes	Yes	Yes	Yes	Yes	No	Yes	6	A
Bankosz et al. (2018b) [25]	Yes	Yes	Yes	Yes	Yes	Yes	Yes	7	A
Iino Yoichi (2017) [26]	Yes	Yes	Yes	Yes	Yes	No	Yes	6	A

Note: ➀ The study design was scientific and rigorous ➁ The data collection strategy is reasonable ➂ The research reports sample response rates ➃ The total representativeness of samples were favorable ➄ The research purpose and method are reasonable ➅ The power of the test was reported ➆ The statistical method was correct.

## Data Availability

Data sharing not applicable No new data were created or analyzed in this study. Data sharing is not applicable to this article.
